# Validation of Maternal Report of Receipt of Iron–Folic Acid Supplementation during Antenatal Care in Rural Southern Nepal

**DOI:** 10.1093/jn/nxab336

**Published:** 2021-09-21

**Authors:** Emily Bryce, Melinda Munos, Tsering Pema Lama, Subarna K Khatry, Steve LeClerq, Joanne Katz

**Affiliations:** Johns Hopkins Bloomberg School of Public Health, Baltimore, MD, USA; Johns Hopkins Bloomberg School of Public Health, Baltimore, MD, USA; Johns Hopkins Bloomberg School of Public Health, Baltimore, MD, USA; Johns Hopkins Bloomberg School of Public Health, Baltimore, MD, USA; Nepal Nutrition Intervention Project–Sarlahi, Kathmandu, Nepal; Johns Hopkins Bloomberg School of Public Health, Baltimore, MD, USA; Nepal Nutrition Intervention Project–Sarlahi, Kathmandu, Nepal; Johns Hopkins Bloomberg School of Public Health, Baltimore, MD, USA

**Keywords:** coverage, antenatal care, measurement, iron–folic acid, validation, Nepal

## Abstract

**Background:**

Coverage of iron–folic acid (IFA) supplementation is a key indicator for tracking programmatic progress within and across countries. However, the validity of maternal report of this information during household surveys has yet to be determined.

**Objectives:**

This study aimed to examine the validity of maternal recall of receipt of IFA supplementation during antenatal care (ANC) and factors associated with accuracy of maternal recall.

**Methods:**

A longitudinal cohort design was employed. The direct observation of the IFA received during all ANC visits at the 5 study health posts served as the “gold standard” to the maternal report of IFA received during the postpartum interview. Individual-level validity was assessed by calculating indicator sensitivity, specificity, and AUC. The inflation factor (IF) measured population-level bias. A multivariable log-binomial model was used to assess factors associated with accurate recall.

**Results:**

The majority (95.8%) of women were observed receiving IFA during pregnancy. Women overreported the number of IFA tablets received compared with what was observed during ANC visits (mean difference: 45 tablets). Maternal report of any IFA receipt was moderate (AUC = 0.60; 95% CI: 0.50, 0.71), and population bias was low (IF = 1.01). However, the individual-level validity was poor across the 7 IFA tablet count categories; the AUC for categories ranged from misleading to moderate. Driven by the trend of maternal overreport, the IF indicated that maternal report drastically underestimated the coverage of lower tablet categories and overestimated the coverage of higher tablet counts. Accuracy of maternal report was not associated with months since last ANC observation nor any maternal characteristics.

**Conclusions:**

Maternal report of the amount of IFA supplementation received during pregnancy produced extremely biased population coverage and performed poorly to moderately for individual-level validity. It is imperative to improve this indicator because it is used in global frameworks and national program planning.

## Introduction

An estimated 38% of pregnant women globally are anemic; half of these cases are attributed to iron deficiency (1). WHO defines anemia during pregnancy as a hemoglobin (Hb) concentration <110 g/L, although in the second trimester this cutoff decreases to a Hb concentration of 105 g/L (2). WHO recommends daily supplementation of 30–60 mg of elemental iron and 400 μg of folic acid during pregnancy to prevent maternal anemia, puerperal sepsis, low birth weight (LBW), and preterm birth (3). Other individual studies have demonstrated an association between iron–folic acid (IFA) supplementation and reductions in postpartum hemorrhage; maternal, perinatal, neonatal, and <5-y mortality; childhood anemia; and improved cognitive development (4–11).

Coverage of IFA supplementation during pregnancy is commonly collected through large population surveys such as the Demographic and Health Survey (DHS). Coverage is defined as the proportion of a population in need of an intervention that receives the intervention (12). The DHS collects data on antenatal care (ANC), including IFA supplementation, through maternal report of services received during a woman's most recent pregnancy. DHS version 7 (DHS7) asks about the most recent live birth in the past 5 y. DHS8, published in June 2020, asks about iron supplementation for the most recent live birth or stillbirth in the past 3 y (13). The 2016 Nepal DHS (DHS7) asks, “During this pregnancy, were you given or did you buy any iron tablets?” and if the women reports yes, it then asks, “During the whole pregnancy, for how many days did you take the tablets?” (14). Using the DH7 parameters, IFA coverage is defined as the proportion of women who had a live birth in the past 5 y who consumed any IFA tablets during that pregnancy.

The policies for the number of days of IFA supplementation during pregnancy varies across countries; examples include >90, >100, and >180 d of IFA consumption during pregnancy (15). The most recent policy in Nepal, National Anemia Strategy 2002, stipulates 180 tablets antenatally and 45 tablets postpartum for a total regimen of 225 tablets. The 2016 coverage estimates in Nepal show that although 90.2% of pregnant women reported receiving or buying any IFA during their last pregnancy, only 42% of women reported consuming ≥180 tablets during the pregnancy. Forty-six percent of pregnant women in Nepal are anemic, indicating that the need for IFA supplementation is great in this population (14).

There is a growing body of evidence examining maternal report of services received during antenatal, labor, and postpartum care and care-seeking for childhood illness that reports a range of indicator validity (16–21). There are limited data of the validity of maternal report of nutrition coverage indicators, including IFA supplementation. The IFA coverage indicator is a core process indicator of the Global Nutrition Monitoring Framework, an indicator for the Countdown to 2030, and is used by countries to inform programming and policies (15, 22). Therefore, it is essential that its measurement is valid.

This study's primary objective was to examine the validity of maternal report of IFA supplementation receipt during ANC and factors associated with accuracy of maternal report. A secondary objective was to examine maternal characteristics associated with receiving or buying IFA from other sources than the government health post.

## Methods

### Study site

This study was conducted from December 2018 to November 2020 in part of the Nepal Nutrition Intervention Project–Sarlahi (NNIPS) study area. In conjunction with our local study team with >30 y of experience conducting research in Sarlahi, 2 municipalities were chosen because of their demographic composition and to limit the bureaucratic permissions required, as Nepal's health system is decentralized to the municipality level. The Sarlahi district is located in the southern Terai region, where it borders the state of Bihar, India. Subsistence farming is the primary economic activity in the district, although income from migratory labor to the Gulf states has become increasingly common. Approximately 60% of the female population in Sarlahi cannot read or write, and 69% are married between the ages of 15 and 19 y (23).

### Study population, design, and data collection

Pregnant women were recruited, consented, and enrolled at 5 public health posts in the district. The health posts were chosen based on ANC case load and geographic location, in terms of accessibility for both the clients and our study staff who had to travel to the posts daily. Women who were attending their first ANC visit who were married, aged ≥15 y, and lived in the study area were eligible. Women are assumed to be married if pregnant and attending ANC, and it would have been culturally inappropriate to ask to confirm marital status, so all women enrolled are assumed to be married. Women who had already attended an ANC visit (including an ultrasound appointment), were aged <15 y, were planning to attend a nonstudy health post or to leave the NNIPS study area while pregnant, or were through 6 mo postpartum were ineligible. Participants were consented during the enrollment visit and again at the postpartum interview.

As noted previously, the DHS asks about IFA consumption during pregnancy. However, establishing a gold standard for consumption would be extremely difficult because this would require observing pregnant women every day. Therefore, the primary objective of the study was to validate maternal report of IFA received during ANC. To accomplish this, we employed a longitudinal cohort design in which the direct observation of providers giving IFA to pregnant women during ANC at the health posts served as the “gold standard” (24). The study observers were trained prior to study implementation. The observer training included videos of mock as well as real ANC visits, in addition to field training with a gold standard observer. Each observer was trained until they reached a certain level of inter- and intraobserver agreement with the videos and the gold standard observer in the field. The direct observation by the study observer was conducted using checklist of 28 items, including 1 IFA-related item, “How many tablets was the woman given?” During the direct observation of the ANC visit, which in the 5 health posts occurred in a single room, the trained study observer would record 000 for zero tablets or a number between 001 and 180.

During the enrollment visit, a demographic questionnaire was administered. At each subsequent ANC visit, the woman was given a brief follow-up questionnaire concerning care-seeking between direct observations. These follow-up questionnaires were used to determine if the woman bought or received IFA between visits (e.g., at a local pharmacy) that we did not observe; for the “gold standard,” it was vital for us to observe all the services received during pregnancy. The direct observation was then compared with maternal report, which was collected ∼6 mo postpartum at the woman's home or *maiti* (parental home). The 6-mo postpartum questionnaire included questions asked in the same language as the 2016 Nepal DHS and about services received at the study health posts specifically. The questionnaire also collected information on socioeconomic status (SES) and pregnancy outcome.

### Analysis

A target sample size of 300 women was established for the overall study based on a conservative 50% coverage for IFA receipt and the assumption that coverage of counseling topics would be lower (14). This allowed for a 95% CI with a width of 0.13 for an AUC equal to 0.50, which is equivalent to a random guess. To account for loss to follow-up, women who went elsewhere for ANC that could not be directly observed, and women who did not have a live birth, the study aimed to enrolled 450 women.

In this analysis, we aimed to validate maternal report of *1*) any IFA receipt and *2*) the number of tablets reported received (**Box 1**). If the woman was observed receiving any IFA tablets (tablet count of ≥1) during ANC observation, this was considered receipt of “any” IFA. We collected the exact number of tablets received and then categorized it into 7 groups; 0, 1 to <30, 30 to <60, 60 to <90, 90 to <120, 120 to <180, and ≥180 tablets.

Box 1 **Two measures of IFA receipt**

*Direct observation/gold standard (“gold standard”)*: The gold standard of the number of IFA tablets received, established by direct observation of each ANC visit at the study health post, during pregnancy.

*Direct observation/gold standard, complete follow-up*: A subset of participants who never reported receiving or buying IFA elsewhere in between direct observations. This was determined using information collected via the follow-up questionnaire at the start of the second and all subsequent direct observations of ANC visits.

*Reported received at study health posts (“reported received”)*: The number of IFA tablets the woman received at the study health posts during her entire pregnancy, as reported by the woman at the postpartum interview. This is the question for the true validation analysis.

The “gold standard” was compared to “reported received” for validation analysis. Using the follow-up questionnaire data, we were able to identify a subcohort of women who never reported receiving or buying IFA between direct observations. This subset represents a more ideal gold standard, where we are more confident that we observed all IFA received during a woman's pregnancy (Box 1). The same validation outcomes were measured in this smaller group of participants as a sensitivity analysis.

The number of tablets observed received compared with reported received was examined by scatterplot. “Don't know” responses in the postpartum follow-up interview were recorded. We constructed 2 × 2 tables to calculate the sensitivity (Se) and specificity (Sp). IFA categories based on a small number of true positive or negative observations that produce estimates with a high degree of uncertainty (95% CIs >15 percentage points) are presented but flagged for readers to interpret with caution. The AUC and the inflation factor (IF) were then calculated to assess validity. Sensitivity, specificity, and AUC measure individual-level validity, and the IF measures population-level validity. The AUC represents the area under a plot of the indicator's sensitivity against (1 – specificity) and is defined as “the probability that the test will correctly classify one positive case and one negative case” (24). Although this measure is commonly used for cutoffs for diagnostic tests, the AUC in this case represents a summary measure of the individual-level validity. An AUC = 0.5 would be comparable to a random guess, and an AUC = 1 would indicate perfect validity. The IF is the ratio of the study coverage (Pr), given the indicator's Se and Sp, to the true coverage (P), based on the gold standard. The study coverage is calculated by the following equation: Pr = P × (Se + Sp – 1) + (1 – Sp) (25). The IF quantifies the degree to which the survey indicator over- or underestimates the true population coverage. An IF between 0.75 and 1.25 indicates low population bias, with 1.00 indicating that the estimate of coverage from the survey is equal to the true coverage (24).

Factors associated with the accuracy of maternal report were examined through a log-binomial bivariate and multivariable regressions (or a Poisson regression if the log-binomial did not converge). The response variable “accuracy” is a dichotomous variable, indicating “accurate” and “inaccurate” responses. A response is accurate if the woman's reported number of IFA tablets received at the health posts falls within the same category (as outlined previously) of the count recorded during direct observation. An inaccurate response is a maternal report of a count outside of the observed count category. Maternal age (age <20 y compared with age ≥20 y), education (none compared with any), parity (nulliparous compared with multiparous), and household wealth were included in the model. The household wealth variable was constructed by summing 11 binary household characteristics (e.g., fuel and drinking water sources) and ownership variables (e.g., number of cattle or motorcycles owned), dividing each woman's total by the number of non-missing variables and separating this proportion into quartiles. Report of time from the last ANC observation was dichotomized to more or less than 12 mo after examining the locally weighted scatterplot smoother (LOWESS) curve. The observed number of tablets the woman received during ANC was also included in the model to examine if women can better report fewer or larger numbers of tablets. The number of observed tablets was classified as 0–60, 60–120, and >120 after the review of the LOWESS curve. Log-binomial bivariate and multivariable regressions were used to examine associations between maternal characteristics and receipt or purchase of IFA elsewhere during ANC. A *P* value <0.05 was considered significant.

All analyses were conducted using Stata version 14.2 (StataCorp).

### Ethical approval

The Institutional Review Board of the Johns Hopkins Bloomberg School of Public Health and the Nepal Health Research Council approved the parent study.

## Results

**FIGURE 1:**
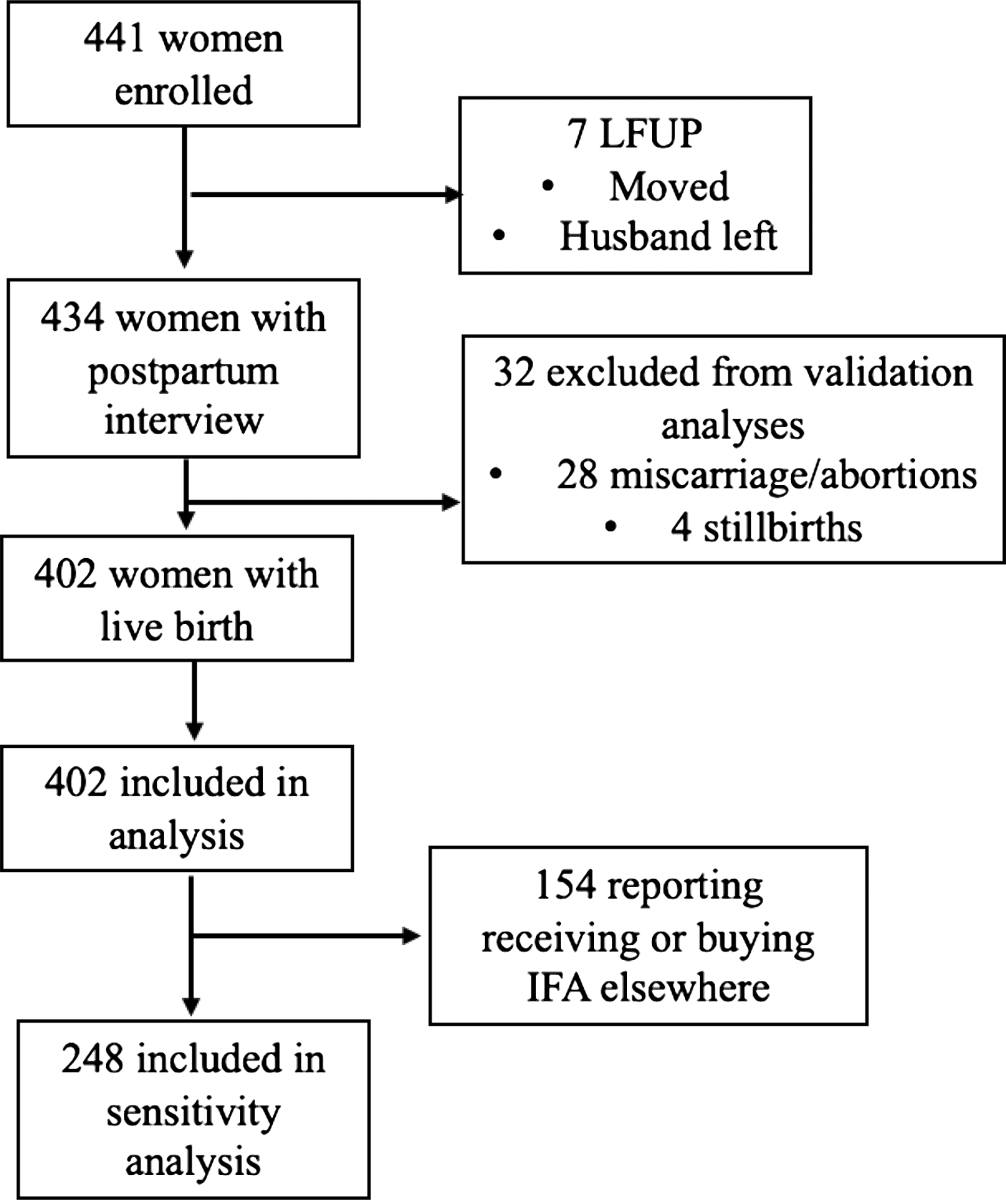
Flowchart of participants. IFA, iron–folic acid; LFUP, lost to follow-up.

A total of 441 women were enrolled and 434 women completed the postpartum interview (**Figure 1**). The 7 women (1.5% of sample) lost to follow-up had moved out of the study area or had life changes, such as divorce, that did not allow the study team to contact them. There were no differences between the women lost to follow-up and those who remained in the study.

There were 278 women (64%) who did not report ever receiving or buying IFA between ANC observations, which includes the 46 women who attended only 1 ANC visit. The maternal characteristics associated with receiving or buying IFA elsewhere in a multivariate model were any years of education (adjusted RR: 1.38; 95% CI: 1.06, 1.81) and primiparity (adjusted RR: 1.41; 95% CI: 1.01, 1.95) (**Supplemental Table 1**).

The average age of the women enrolled was 22.5 y, ranging from 16 to 41 y (**Table 1**). The number of ANC visits observed per woman ranged from 1 to 14, with the average number of visits observed equal to 4.5 visits. The average SES composite score was 6.3 of the possible 11 points, indicating low ownership. Overall, 59.6% of the women reported 0 y of education. The majority of participants had a live birth; there were 28 miscarriages/abortions (6%) and 4 stillbirths (<1%).

**TABLE 1:** Characteristics of enrolled participants^1^

	Observed all IFA receipt (*n* = 278)		Received or bought IFA between observations (*n* = 156)		Two-sample *t*-test *P* value	Total (*n* = 434)	
Characteristic	Mean ± SD	Range	Mean ± SD	Range		Mean ± SD	Range
Woman's age, y	22.7 ± 4.3	16–41	22.2 ± 3.9	16–35	0.186	22.5 ± 4.2	16–41
Total no. of ANC visits observed	3.8 ± 2.4	1–13	5.6 ± 2.3	2–14	<0.01	4.5 ± 2.5	1–14
No. of months between last ANC observation and postpartum interview	10.8 ± 3.3	3–22	9.4 ± 2.6	3–18	<0.01	10.3 ± 3.2	3–22
	Observed all IFA receipt (*n* = 278)		Received or bought IFA between observations (*n* = 156)		Chi-square	Total (*n* = 434)	
	*n* (%)		*n* (%)		*P* value	*n* (%)	
Most recent pregnancy outcome							
Miscarriage/abortion	28 (10.1)		0 (0.0)		<0.01	28 (6.5)	
Stillbirth	2 (0.7)		2 (1.3)			4 (0.9)	
≥1 live birth	248 (89.2)		154 (98.7)			402 (92.6)	
Four quantiles of SES							
1	118 (42.5)		49 (31.4)		0.15	167 (38.5)	
2	45 (16.2)		29 (18.6)			74 (17.1)	
3	80 (28.8)		52 (33.3)			132 (30.4)	
4	35 (12.5)		26 (16.7)			61 (14.0)	
Is this the woman's first pregnancy?							
No	202 (72.7)		96 (61.5)		<0.05	298 (68.7)	
Yes	76 (27.3)		60 (38.5)			136 (31.3)	
Had the woman received any schooling?							
No	183 (65.8)		76 (48.7)		<0.01	259 (59.7)	
Yes	95 (34.2)		80 (51.3)			175 (40.3)	
Trimester at enrollment, mo							
1–3	114 (41.0)		76 (48.7)		0.14	190 (43.8)	
4–6	157 (56.5)		79 (50.7)			236 (54.4)	
7–9	7 (2.5)		1 (0.6)			8 (1.8)	

1 ANC, antenatal care; IFA, iron–folic acid; SES, socioeconomic status.

**FIGURE 2:**
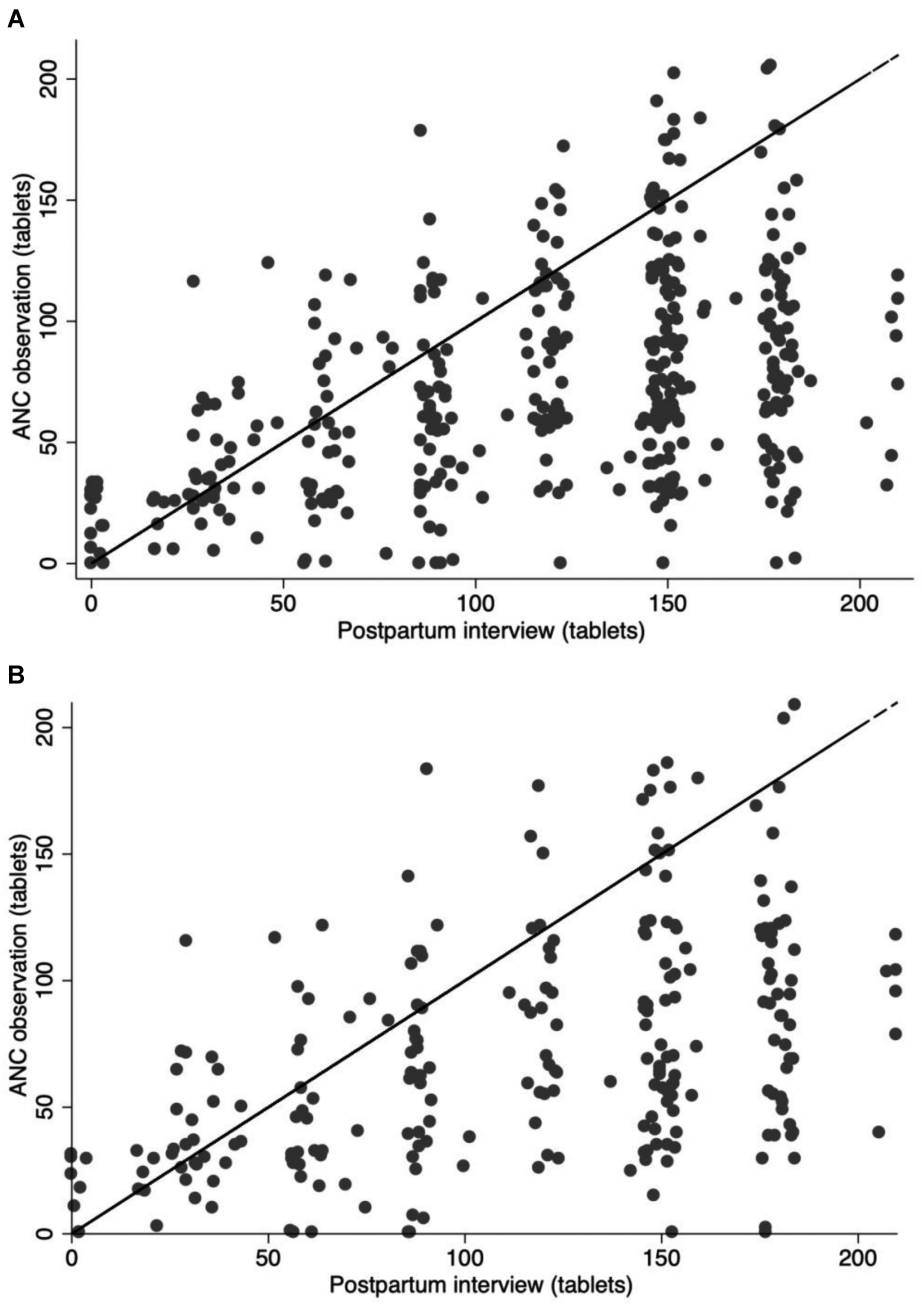
Comparing the number of tablets observed received during ANC visits with maternal reported received at postpartum interview in (A) entire cohort (*n* = 402) and (B) subcohort with all IFA receipt observed (*n* = 248). ANC, antenatal care; IFA, iron–folic acid.

The scatterplots in **Figure 2** illustrate the differences in the “gold standard” and the “reported received.” In the entire cohort (Figure 2A), the mean number of tablets observed received was 73.1 tablets (SD = 43.8), compared with the mean reported received of 118.5 tablets (SD = 53.3). In the subcohort (Figure 2B), the mean values for observed received and reported received were 71.5 tablets (SD = 45.5) and 115.4 tablets (SD = 55.7), respectively. There was a trend of overreporting at the postpartum interview compared with what was observed during ANC. In fact, 72.6% of women overreported the count of IFA tablets received, on average by ∼70 tablets (μ: 69.2; range: 65.2–72.8) (**Supplemental Figure 1**). Reported numbers tended to be heaped, whereas observed number of tablets were more evenly spread out from 0 to >200.

**FIGURE 3:**
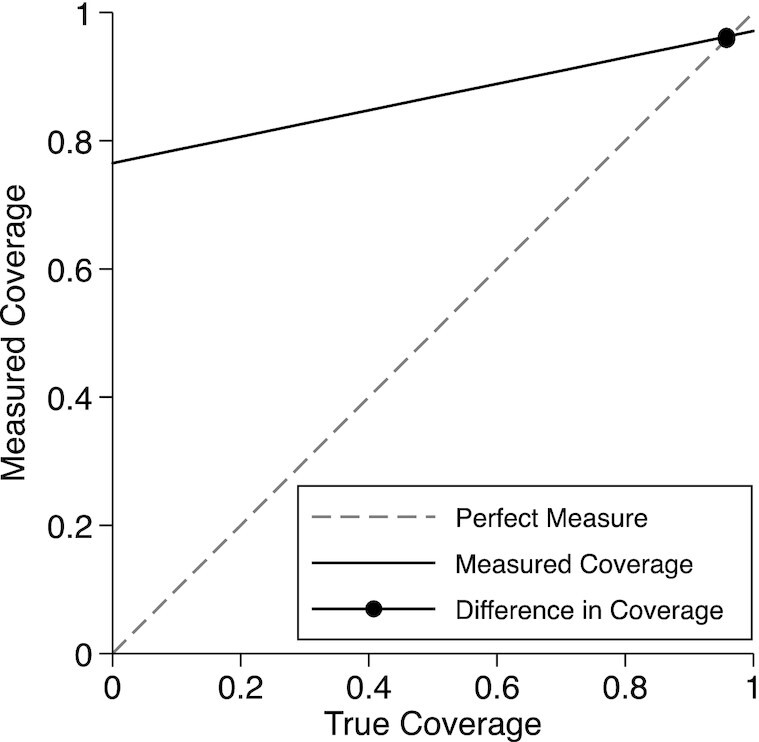
True coverage compared with measured coverage for receipt of any IFA during antenatal care. IFA, iron–folic acid.

The validation analyses were conducted only among women with a live birth because this is how the most recent DHS in Nepal was conducted. The analytical cohort included 402 women with live births, for 248 of whom all IFA receipt was observed. The validation results comparing the “gold standard” to “reported received” are presented in **Table 2**. There were 0 “Don't know” responses for the report of IFA during the postpartum interview. Validation of any IFA receipt had moderate individual accuracy (AUC: 0.60; 95% CI: 0.50, 0.71) and low population bias (IF: 1.01). Specificity was low, meaning that women who were not observed receiving IFA often reported receiving IFA at the postpartum interview. Although there was low population bias in this population, **Figure 3** shows that at a lower coverage, the survey question will overestimate the true coverage.

**TABLE 2:** Validation of maternal report of IFA supplementation received at 5 study health posts, among women with live births (*n* = 402)^1^

“Gold standard” vs. “reported received”	Sensitivity (95% CI), %	Specificity (95% CI), %	AUC (95% CI)	“True” coverage (95% CI), %	Estimated survey coverage, %	Inflation factor
Receipt of any IFA	97.1 (94.9, 98.6)	23.5^2^ (6.8, 49.9)	0.60^2^ (0.50, 0.71)	95.8 (93.3, 97.5)	96.2	1.01
No. of IFA tablets						
0	23.5^2^ (6.8, 49.9)	97.1 (94.9, 98.5)	0.60^2^ (0.50, 0.71)	4.22 (2.5, 6.7)	3.8	0.89
1 to <30	16.7^2^ (4.7, 37.4)	99.2 (97.7, 99.8)	0.58^2^ (0.50, 0.66)	5.9 (3.9, 8.8)	1.7	0.29
30 to <60	18.1 (11.3, 26.8)	94.6 (91.4, 96.9)	0.56 (0.52, 0.60)	26.1 (21.9, 30.7)	8.7	0.33
60 to <90	5.6 (2.1, 11.7)	89.1 (84.9, 92.4)	0.47 (0.45, 0.50)	26.9 (22.6, 31.5)	9.5	0.35
90 to <120	16.2 (8.4, 27.1)	86.5 (82.4, 90.0)	0.51 (0.47, 0.56)	16.0 (13.4, 20.9)	13.9	0.87
120 to <180	66.2 (53.7, 77.2)	61.7 (56.2, 66.9)	0.64 (0.58, 0.70)	16.9 (13.4, 20.9)	43.0	2.55
≥180	33.3^2^ (9.9, 65.1)	81.0^2^ (76.8, 84.8)	0.57^2^ (0.43, 0.71)	2.9 (1.6, 5.2)	19.4	6.69

1 IFA, iron–folic acid.

2 Indicates uncertainty around this point estimate because a small number of true positives or negatives resulted in an estimate with a 95% CI >15%.

The validity of maternal report of the number of IFA tablets was poor across all the categories of true tablet counts, or the counts measured by direct observation. For the majority of categories, the AUC was between 0.47 and 0.58, indicating a performance that was at worst misleading and at best barely better than a random guess. The exception was report of 120 to <180 tablets, which had an AUC = 0.64 (95% CI: 0.58, 0.70). At the lower values of the true tablet counts, the inflation factor showed a greater underestimation of the coverage and high population-level bias, driven by the maternal overreporting trend outlined in Figure 3. As the true tablet counts increased to ≥120, the inflation factor indicated an overestimation of the coverage and, again, high population-level bias.

The “gold standard” compared with “reported received” validation analyses were also run in the entire cohort, including women who had miscarriages/abortions (*n* = 28) and stillbirths (*n* = 4). The inclusion of these women improved the validity of maternal report of receipt of any IFA (**Supplemental Table 2**). The AUC in this population was equal to 0.75 (95% CI: 0.67, 0.83), which is considered high individual-level validity. The specificity increased as well to 52.8% (95% CI: 35.5, 69.9%). The tablet-count validation results were slightly improved in the entire cohort, although this did not qualitatively change the interpretation. The women with live births had a significantly higher average number of visits and observed number of tablets received compared with the women with adverse pregnancy outcomes (μ_1_ = 4.65 visits compared with μ_2_ = 1.97 visits, *P* < 0.01; and μ_1_ = 73.1 tablets compared with μ_2_ = 16.9 tablets, *P* < 0.01). In fact, 50% of women with adverse pregnancy outcomes had only 1 ANC visit, and 59.4% of these women received 0 tablets, which may explain some of the differences in specificity and AUC between the 2 analyses.

Restricting the analysis to the women who never reported receiving or buying IFA between observations in the entire cohort for the sensitivity analysis did not change the validity results (**Table 3**). Sensitivity improved slightly for the restricted group, although it did not change the AUC or the trend for population-level bias across tablet count categories.

**TABLE 3:** Sensitivity analysis: validation of maternal recall of IFA supplementation received at 5 study health posts among women with live births and complete follow-up (*n* = 248)^1^

“Gold standard” vs. “reported received”	Sensitivity (95% CI), %	Specificity (95% CI), %	AUC (95% CI)	“True” coverage (95% CI), %	Estimated survey coverage, %	Inflation factor
Receipt of any IFA	97.5 (94.5, 99.0)	16.7^2^ (2.1, 48.4)	0.57^2^ (0.46, 0.68)	95.2 (91.7, 97.5)	96.8	1.02
No. of IFA tablets						
0	16.7^2^ (2.1, 48.4)	97.5 (94.5, 99.0)	0.57^2^ (0.46, 0.68)	4.8 (2.5, 8.3)	3.2	0.66
1 to <30	20.0^2^ (4.3, 48.1)	98.7 (96.3, 99.7)	0.59^2^ (0.49, 0.70)	6.0 (3.4, 9.8)	2.4	0.40
30 to <60	20.3 (11.8, 21.2)	93.7 (88.9, 96.8)	0.57 (0.52, 0.62)	29.8 (24.2, 35.9)	10.5	0.35
60 to <90	7.1 (2.0, 17.3)	87.0 (82.4, 91.4)	0.47 (0.43, 0.51)	22.6 (17.5, 28.3)	11.7	0.52
90 to <120	16.3^2^ (6.8, 30.7)	86.3 (80.9, 90.7)	0.51 (0.45, 0.57)	17.3 (12.8, 22.6)	14.1	0.82
120 to <180	53.8^2^ (37.2, 69.9)	66.0 (59.2, 72.4)	0.60 (0.51, 0.68)	15.7 (11.4, 20.9)	37.1	2.36
≥180	33.3^2^ (7.5, 70.1)	79.5 (73.8, 84.4)	0.56^2^ (0.40, 0.73)	3.6 (1.7, 6.8)	21.0	5.82

1 IFA, iron–folic acid.

2 Indicates uncertainty around this point estimate because a small number of true positives or negatives resulted in an estimate with a 95% CI >15%.

There were 93 women (23.1%) who accurately reported the number of tablets within the 7 defined categories. Only 9.2% of women reported an exact match of tablets observed and reported received (data not shown). There were no maternal characteristics associated with accurate report of number of IFA tablets received at the 5 study posts (**Table 4**). The number of months since the last ANC observation also did not have a significant association with accuracy; the unadjusted risk showed that a lag of >12 mo is associated with a slight decrease in the accuracy RR, but after adjustment for other variables in the model, the RR increased to 1.29 (95% CI: 0.73, 2.28). The strongest association with an accurate response was with an observed count of IFA tablet receipt >120 compared with a count between 0 and 60 tablets (adjusted RR: 3.68; 95% CI: 2.21, 6.13). Those who were observed receiving 60 to <120 tablets were almost half as likely to report this information accurately compared with those receiving 0 and 60 tables, although this association was only significant in the bivariate analyses.

**TABLE 4:** Maternal characteristics associated with accuracy^1^

	*n* (%)	Unadjusted RR (95% CI)	Adjusted RR (95% CI)
Maternal characteristics			
Any education	162 (40.3)	1.02 (0.71, 1.47)	1.05 (0.67, 1.64)
No. of previous live birth	126 (31.3)	0.76 (0.50, 1.15)	0.70 (0.40, 1.22)
Age <20 y	165 (41.0)	0.91 (0.63, 1.31)	1.33 (0.80, 2.21)
SES quartiles (ref: first)			
2	71 (17.6)	0.95 (0.57, 1.60)	1.04 (0.57, 1.90)
3	126 (31.3)	1.01 (0.66, 1.54)	0.89 (0.54, 1.47)
4	57 (14.2)	0.89 (0.50, 1.59)	0.99 (0.50, 2.00)
Study factors			
>1 y since last ANC observation	76 (18.9)	0.96 (0.60, 1.52)	1.29 (0.73, 2.28)
No. of IFA tablets (ref: 0 to <60)			
60 to <120	175 (43.8)	0.52 (0.30, 0.92)^2^	0.55 (0.30, 1.02)
≥120	80 (19.9)	3.31 (2.26, 4.86)^3^	3.68 (2.21, 6.13)^3^

1 ANC, antenatal care; IFA, iron–folic acid; SES, socioeconomic status.

2 *P* < 0.05.

3 *P* < 0.01.

## Discussion

This study estimated the validity of maternal report of IFA supplementation receipt during pregnancy, including the report of the number of tablets received. To our knowledge, this is the first study to assess the validity of maternal report of the number of IFA tablets received. We found that report of any IFA supplementation during ANC had moderate individual-level validity and low population bias in a population with high coverage. However, at the population level, maternal report of the number of tablets received underestimated the true coverage at the lower range of tablet counts and greatly overestimated the true coverage at the high range of tablet counts. No maternal characteristics included in the analysis were associated with accuracy of maternal report.

We observed 95.8% of women receiving IFA during ANC. This is greater than the 2016 Nepal DHS estimate of 86.7% for Province 2, where the study site is located (14). This difference could be attributed to the fact that the DHS includes women who never attended ANC, whereas in our study all women had ≥1 ANC visit. We observed 2.8% of women receiving ≥180 IFA tablets. In comparison, the 2016 DHS reports 42% of women consuming IFA tablets for ≥180 days (14). In Province 2, the proportion was lower at 28% but is still nearly 4 times greater than what was observed during our study. However, the DHS estimate is likely an overestimation because, as we have shown in this analysis, maternal report of tablet counts >120 tended to greatly overestimate the coverage. A potential reason for the overreporting could be social desirability bias—the tendency of individuals to provide what they think the interviewer and society would consider a favorable response (26). In this case, women may want to appear to the interviewer that they received more tablets than they did in order to appear to have received more complete care. This may also explain why women who received a greater number of tablets were >3 times more likely to report accurately; the women who received a greater number of tablets did not feel compelled to overreport. In addition, another study demonstrated that interventions with high coverage tend to be overreported, driven by the logic that women assume they should have received the interventions (27).

One previous study examined the validity of maternal report of any IFA receipt using data collected from 9 service provision assessments (SPAs) and a small sample from a previous study at the same site as our study (21). The SPA validation analyses produced a sensitivity of 88.7%, specificity of 79.3%, an AUC >0.70 (their cutoff for high individual-level validity), and low population bias. However, these results are from exit interviews immediately following an ANC visit, so they are not necessarily comparable to those presented in this article. The validation results from the previous study in our study area are much more comparable: sensitivity = 86.1%, specificity = 34.3%, AUC = 0.60, and IF = 1.43. This study was population based and included women who did not attend ANC but who were provided supplements as part of the study. Their report period ranged from 1 to 2 y, which is slightly longer than ours (range: 1–22 m; mean report time: 9.1 months from date of pregnancy outcome). In addition, their coverage of IFA receipt was much lower than ours (53.7% compared with 95.8%, respectively), which resulted in higher population bias (IF = 1.43 compared with IF = 1.0, respectively), despite the lower specificity in our study.

To our knowledge, this is the first study to examine the validity of maternal report of the number of tablets received during pregnancy. Maternal report of birth weight has been examined in validity studies, which is another instance of validating a numerical response rather than a “yes or no” response. A study conducted in Taiwan examined maternal report of birth weight and found that although women were able to accurately report whether their infant was LBW, the accuracy for reporting the specific weight category was low (15.9%) and women tended to overreport their infant's birth weight (28). This is slightly lower than the 21.8% of women accurately reporting categories of IFA receipt in our study. A study at our same site in Nepal had low sensitivity for maternal report of birth weight to classify LBW and of length of gestation to classify preterm birth (19). This is similar to the low sensitivity for classifying categories of IFA tablets received in our study population.

No associations were found between maternal characteristics in a multivariate model and accuracy of report of number of IFA tablets received in this study, which is similar to findings from other validation studies that report no patterns of association between maternal SES, age, parity, or education and accuracy (29, 30). In contrast, other studies examining maternal report of LBW have reported parity and maternal education to be associated with accuracy (19, 31). There was no observed association with accuracy and length of report period, which is consistent with other studies’ findings (19, 28).

A woman having any years of education was significantly associated with receiving IFA elsewhere between observations. Women with higher education have been shown to be more likely to access health services and receive higher quality care (32, 33). It is possible that women with higher education were aware of the benefits of IFA during pregnancy and thus sought its receipt outside of the health facilities, where stocks can be limited. A woman being pregnant for the first time was also more likely to receive IFA between visits in our study. Higher parity is associated with decreased ANC attendance (34) and lower quality ANC (32). Therefore, women with higher parity may also be less likely to seek additional services outside of the health facility.

Given the frequent use of the consumption indicator in global nutrition tracking and national-level policy and programs, it is important to consider how the indicator is defined and measured. There are multiple factors to consider when defining the coverage indicator for the number of tablets consumed and/or received during pregnancy. First, the policies for the amount of IFA consumed during pregnancy differ across countries (15), so there is no standard “adequate” amount from a policy perspective. Second, the prevalence of iron-deficient anemia varies by country (1), representing a difference in need from a biological perspective. Finally, the mean number of ANC visits varies by country (35), meaning from a programmatic perspective there is no standard number of opportunities to provide IFA during ANC. The results of the study suggest against using household surveys to measure the coverage of the amount of IFA received during pregnancy because the amount reported received is greatly overestimated. However, there is not a clear alternative. Routine health information systems, including electronic health records, are generally rather weak in low- and middle-income countries and often focus on aggregated rather than individual data (36). Their use for measurement of IFA receipt is further complicated by the fact that many women received IFA from other sources, such as pharmacies, which would not be captured by these systems. Furthermore, if functional and available, these systems would capture receipt, not consumption, of IFA. Further consideration is needed to decide how to best define and measure the amount of IFA received or consumed and under which circumstances reporting this indicator would be most useful and accurate. In addition, further research in private facility settings, semiurban or urban areas with higher education levels, and more varied IFA coverage would be beneficial to best inform the indicator development and its measurement.

A strength of this study is the use of direct observation by trained study observers as the gold standard, which is the preferred standard for validation studies. Another strength is that although our 6-mo study report period is shorter than the DHS's 3- or 5-y report period, it is much longer than those of other validation studies that have used exit interviews to measure maternal report accuracy. A limitation of this study is that we observed IFA receipt only at our 5 government health posts. This presents 2 issues: our sample included only women who sought ANC, and we were unable to observe all sources of IFA receipt for all the women in the study. This may mean that our findings may not be generalizable to women who do not attend ANC at all or who attend private facilities. We attempted to address the inability to observe all IFA receipt through the use of follow-up questionnaires to identify women who did not receive or buy IFA between visits. However, this approach does rely on reports by the women that they did not go elsewhere to obtain IFA. The potential of an observer effect is a third limitation, whereby providers alter their care because of the presence of our study staff at the facility. To mitigate this possibility, during the consent process, the providers were informed that the study staff were not medically trained (and therefore would not know if the care they observed was correct or not) and would not report any findings to the provider's superiors. Furthermore, the study observers were stationed in the facility every business day for more than 1 y, so we hope that if present initially, the observer effect lessened over time. Another limitation of this study was the reliance on accurate reporting of observed IFA receipt and number of tablets given during ANC visits by the study observers in the health facilities. We believe this limitation was reduced by the rigorous training of observers.

In conclusion, the use of maternal report of any IFA receipt during pregnancy had moderate individual-level validity and low population bias, meaning that the use of this indicator in surveys to measure any IFA received during pregnancy accurately estimates the population coverage. However, maternal report of the number of IFA tablets received produced extremely biased population coverage and performed comparably to or worse than a random guess for individual-level validity. Additional research is needed to assess maternal report of IFA receipt in other settings with more variable IFA coverage levels and to further elucidate reasons for inaccurate reporting of IFA tablet counts to improve the indicator for future use.

## Supplementary Material

nxab336_Supplemental_File

## Data Availability

Data described in the manuscript, code book, and analytic code will be made available upon request pending approval.

## Notes

This research was funded by the Bill & Melinda Gates Foundation. The funders had no role in the design or implementation of the study, nor the analysis or interpretation of the data. MM and JK were the funding recipients. This work was supported, in whole or in part, by the Bill & Melinda Gates Foundation (OPP1172551). Under the grant conditions of the Foundation, a Creative Commons Attribution 4.0 Generic License has already been assigned to the Author Accepted Manuscript version that might arise from this submission.

Author disclosures: The authors report no conflicts of interest.

Supplemental Tables 1 and 2 and Supplemental Figure 1 are available from the “Supplementary data” link in the online posting of the article and from the same link in the online table of contents at https://academic.oup.com/jn/.

Abbreviations used: ANC, antenatal care; DHS, Demographic and Health Survey; Hb, hemoglobin; IF, inflation factor; IFA, iron–folic acid; LBW, low birth weight; LOWESS, locally weighted scatterplot smoother; NNIPS, Nepal Nutrition Intervention Project–Sarlahi; Se, sensitivity; SES, socioeconomic status; Sp, specificity; SPA, service provision assessments.

## References

[1] Stevens GA, Finucane MM, De-Regil LM, Paciorek CJ, Flaxman SR, Branca F, Pena-Rosas JP, Bhutta ZA, Ezzati M; Nutrition Impact Model Study Group. Global, regional, and national trends in haemoglobin concentration and prevalence of total and severe anaemia in children and pregnant and non-pregnant women for 1995–2011: a systematic analysis of population-representative data. Lancet Global Health. 2013;1(1):e16–25. 25103581 10.1016/S2214-109X(13)70001-9 PMC4547326

[2] WHO. Haemoglobin concentrations for the diagnosis of anaemia and assessment of severity. Geneva (Switzerland): WHO; 2011.

[3] WHO. WHO recommendations on antenatal care for a positive pregnancy experience. Geneva (Switzerland): WHO; 2016. 28079998

[4] Kavle JA, Stoltzfus RJ, Witter F, Tielsch JM, Khalfan SS, Caulfield LE. Association between anaemia during pregnancy and blood loss at and after delivery among women with vaginal births in Pemba Island, Zanzibar, Tanzania. J Health Popul Nutr. 2008;26(2):232–40. 18686556 PMC2740668

[5] Black RE, Victora CG, Walker SP, Bhutta ZA, Christian P, de Onis M, Ezzati M, Grantham-McGregor S, Katz J, Martorell R et al. Maternal and child undernutrition and overweight in low-income and middle-income countries. Lancet North Am Ed. 2013;382(9890):427–51. 10.1016/S0140-6736(13)60937-X 23746772

[6] Christian P, Murray-Kolb LE, Khatry SK, Katz J, Schaefer BA, Cole PM, Leclerq SC, Tielsch JM. Prenatal micronutrient supplementation and intellectual and motor function in early school-aged children in Nepal. JAMA. 2010;304(24):2716–23. 21177506 10.1001/jama.2010.1861

[7] Christian P, Stewart CP, LeClerq SC, Wu L, Katz J, West KP, Khatry SK. Antenatal and postnatal iron supplementation and childhood mortality in rural Nepal: a prospective follow-up in a randomized, controlled community trial. Am J Epidemiol. 2009;170(9):1127–36. 19778983 10.1093/aje/kwp253 PMC2781740

[8] Christian P, West KP, Khatry SK, Leclerq SC, Pradhan EK, Katz J, Shrestha SR, Sommer A. Effects of maternal micronutrient supplementation on fetal loss and infant mortality: a cluster-randomized trial in Nepal. Am J Clin Nutr. 2003;78(6):1194–202. 14668283 10.1093/ajcn/78.6.1194

[9] Daru J, Zamora J, Fernandez-Felix BM, Vogel J, Oladapo OT, Morisaki N, Tuncalp O, Torloni MR, Mittal S, Jayaratne K et al. Risk of maternal mortality in women with severe anaemia during pregnancy and post partum: a multilevel analysis. Lancet Global Health. 2018;6(5):e548–54. 29571592 10.1016/S2214-109X(18)30078-0

[10] Nisar YB, Dibley MJ, Mebrahtu S, Paudyal N, Devkota M. Antenatal iron–folic acid supplementation reduces neonatal and under-5 mortality in Nepal. J Nutr. 2015;145(8):1873–83. 26136588 10.3945/jn.114.206565

[11] Nisar YB, Dibley MJ. Earlier initiation and use of a greater number of iron–folic acid supplements during pregnancy prevents early neonatal deaths in Nepal and Pakistan. PLoS One. 2014;9(11):e112446. 25398011 10.1371/journal.pone.0112446 PMC4232391

[12] Requejo JH, Newby H, Bryce J. Measuring coverage in MNCH: challenges and opportunities in the selection of coverage indicators for global monitoring. PLoS Med. 2013;10(5):e1001416. 23667336 10.1371/journal.pmed.1001416 PMC3646210

[13] The DHS Program. Demographic and Health Surveys. [Internet]. 2021. [Accessed 2021 Apr 10]. Available from: https://dhsprogram.com.

[14] Nepal Ministry of Health. Nepal Demographic and Health Survey 2016. Kathmandu (Nepal): Nepal Ministry of Health; 2017.

[15] WHO. Developing and validating an iron and folic acid supplementation indicator for tracking progress towards global nutrition monitoring framework targets. Geneva (Switzerland): WHO; 2018.

[16] Blanc AK, Warren C, McCarthy KJ, Kimani J, Ndwiga C, RamaRao S. Assessing the validity of indicators of the quality of maternal and newborn health care in Kenya. J Global Health. 2016;6(1):010405. 10.7189/jogh.06.010405 PMC4871064 27231541

[17] McCarthy KJ, Blanc AK, Warren CE, Kimani J, Mdawida B, Ndwidga C. Can surveys of women accurately track indicators of maternal and newborn care? A validity and reliability study in Kenya. J Global Health. 2016;6(2):020502. 10.7189/jogh.06.020502 PMC5012235 27606061

[18] Stanton CK, Rawlins B, Drake M, Dos Anjos M, Cantor D, Chongo L, Chavane L, da Luz Vaz M, Ricca J. Measuring coverage in MNCH: testing the validity of women's self-report of key maternal and newborn health interventions during the peripartum period in Mozambique. PLoS One. 2013;8(5):e60694. 23667427 10.1371/journal.pone.0060694 PMC3646219

[19] Chang KT, Mullany LC, Khatry SK, LeClerq SC, Munos MK, Katz J. Validation of maternal reports for low birthweight and preterm birth indicators in rural Nepal. J Global Health. 2018;8(1):010604. 10.7189/jogh.08.010604 PMC5997365 29899981

[20] Liu L, Li M, Yang L, Ju L, Tan B, Walker N, Bryce J, Campbell H, Black RE, Guo Y. Measuring coverage in MNCH: a validation study linking population survey derived coverage to maternal, newborn, and child health care records in rural China. PLoS One. 2013;8(5):e60762. 23667429 10.1371/journal.pone.0060762 PMC3646215

[21] Kanyangarara M, Katz J, Munos MK, Khatry SK, Mullany LC, Walker N. Validity of self-reported receipt of iron supplements during pregnancy: implications for coverage measurement. BMC Pregnancy Childbirth. 2019;19(1):113. 30940114 10.1186/s12884-019-2247-1 PMC6446307

[22] Countdown to 2030 Collaboration. Countdown to 2030: tracking progress towards universal coverage for reproductive, maternal, newborn, and child health. Lancet North Am Ed. 2018;391(10129):1538–48. 10.1016/S0140-6736(18)30104-1 29395268

[23] Central Bureau of Statistics, Government of Nepal. National Population and Housing Census 2011. Kathmandu (Nepal): Central Bureau of Statistics, Government of Nepal; 2012.

[24] Munos MK, Blanc AK, Carter ED, Eisele TP, Gesuale S, Katz J, Marchant T, Stanton CK, Campbell H. Improving coverage measurement: validation studies for population-based intervention coverage indicators: design, analysis, and interpretation. J Global Health. 2018;8(2):020804. 10.7189/jogh.08.020804 PMC6126515 30202519

[25] Vecchio TJ . Predictive value of a single diagnostic test in unselected populations. N Engl J Med. 1966;274(21):1171–3. 5934954 10.1056/NEJM196605262742104

[26] Fisher RK, Katz JE. Social desirability bias and the validity of self-reported values. Psychology Marketing. 2000;17(2):105–20.

[27] Carter ED, Chang KT, Mullany LC, Khatry SK, LeClerq SC, Munos MK, Katz J. Reliability of maternal recall of delivery and immediate newborn care indicators in Sarlahi. BMC Pregnancy Childbirth. 2021;21(1):82. 33494712 10.1186/s12884-021-03547-5 PMC7831166

[28] Li CY, Wei JN, Lu TH, Chuang LM, Sung FC. Mothers tended to overreport categorical infant birth weight of their children. J Clin Epidemiol. 2006;59(12):1319–25. 17098575 10.1016/j.jclinepi.2006.02.018

[29] Carter ED, Ndhlovu M, Munos M, Nkhama E, Katz J, Eisele TP. Validity of maternal report of care-seeking for childhood illness. J Global Health. 2018;8(1):010602. 10.7189/jogh.08.010602 PMC5854307 29619212

[30] McCarthy KJ, Blanc AK, Warren CE, Mdawida B. Women's recall of maternal and newborn interventions received in the postnatal period: a validity study in Kenya and Swaziland. J Global Health. 2018;8(1):010605. 10.7189/jogh.08.010605 PMC5983915 29904605

[31] Boeke CE, Marin C, Oliveros H, Mora-Plazas M, Agudelo-Canas S, Villamor E. Validity of maternal birthweight recall among Colombian children. Matern Child Health J. 2012;16(4):753–9. 21516299 10.1007/s10995-011-0803-z

[32] Joshi C, Torvaldsen S, Hodgson R, Hayen A. Factors associated with the use and quality of antenatal care in Nepal: a population-based study using the demographic and health survey data. BMC Pregnancy Childbirth. 2014;14(1):94. 24589139 10.1186/1471-2393-14-94 PMC3943993

[33] Greenaway ES, Leon J, Baker DP. Understanding the association between maternal education and use of health services in Ghana: exploring the role of health knowledge. J Biosoc Sci. 2012;44(6):733–47. 22377424 10.1017/S0021932012000041 PMC3590019

[34] Simkhada B, Teijlingen ER, Porter M, Simkhada P. Factors affecting the utilization of antenatal care in developing countries: systematic review of the literature. J Adv Nurs. 2008;61(3):244–60. 18197860 10.1111/j.1365-2648.2007.04532.x

[35] Benova L, Tuncalp O, Moran AC, Campbell OMR. Not just a number: examining coverage and content of antenatal care in low-income and middle-income countries. BMJ Global Health. 2018;3(2):e000779. 10.1136/bmjgh-2018-000779 PMC5898334 29662698

[36] Venkateswaran M, Morkrid K, Abu Khader K, Awwad T, Friberg IK, Ghanem B, Hijaz T, Froen JF. Comparing individual-level clinical data from antenatal records with routine health information systems indicators for antenatal care in the West Bank: a cross-sectional study. PLoS One. 2018;13(11):e0207813. 30481201 10.1371/journal.pone.0207813 PMC6258527

