# Supplementary material for: Validation of Maternal Report of Receipt of Iron–Folic Acid Supplementation during Antenatal Care in Rural Southern Nepal
Source: J Nutr. 2021 Sep 21;152(1):310–8. doi: 10.1093/jn/nxab336 (PMC8754516; doi:10.1093/jn/nxab336)
Supplement: nxab336_Supplemental_File [file nxab336_supplemental_file.pdf]

Validation of Maternal Report of Iron Folic-Acid Supplementation During Antenatal Care in Rural Southern Nepal. Emily Bryce. Online Supplementary Material.

Supplementary Table 1. Factors associated with receipt or purchase of IFA elsewhere between direct observations[1]

	N (%)	RR	95% CI	Adjusted RR	95% CI
Maternal characteristics					
Any education	175 (40.3%)	1.52[3]	1.19 to 1.94	1.38[2]	1.06 to 1.81
First pregnancy	136 (31.3%)	1.36[2]	1.06 to 1.74	1.41[2]	1.01 to 1.95
Age < 20	174 (3.78%)	1.12	0.87 to 1.45	0.82	0.59 to 1.13
Presented for ANC in first trimester	190 (43.8%)	1.30[2]	1.01 to 1.66	1.23	0.97 to 1.57
SES quartiles (ref: first)					
2	74 (17.1%)	1.29	0.89 to 1.86	1.21	0.84 to 1.73
3	132 (30.4%)	1.30	0.95 to 1.78	1.15	0.83 to 1.59
4	61 (14.1%)	1.44	0.99 to 2.08	1.14	0.77 to 1.67

1- ANC, Antenatal Care; IFA, Iron Folic-Acid; RR, Relative Risk; SES, Socioeconomic Status

2- $p<0.05$

3- $p<0.01$

Supplementary Table 2. Validation of maternal recall of IFA receipt during pregnancy (N=434). Includes all birth outcomes (live birth, miscarriage, abortion, stillbirth)[1]

Gold standard= ANC Observation Comparison= Report of # of tablets from 5 study HP, specifically	Sensitivity (95% CI), %	Specificity (95% CI), %	AUC (95% CI)	"True" coverage (95% CI), %	Estimated survey coverage, %	Inflation factor
Receipt of any IFA	96.9 (94.8-98.4)	52.8[2] (35.5-69.6)	0.75[2] (0.67-0.83)	91.7 (88.7-94.1)	92.8%	1.01
Number of IFA tablets						
0	52.8 (35.5-69.6)	96.9 (94.8-98.4)	0.75 (0.67-0.83)	8.3 (5.9-11.3)	7.2%	0.87
1 to < 30	15.4[2] (4.4-34.9)	97.8 (95.8-98.9)	0.57[2] (0.49-0.64)	5.9 (3.9-8.7)	3.0%	0.50
30 to < 60	19.6 (12.7-28.2)	94.4 (91.3-96.7)	0.57 (0.53-0.61)	25.8 (21.7-30.2)	9.2%	0.36
60 to < 90	6.3 (2.6-12.6)	90.1 (86.3-93.1)	0.48 (0.45-0.51)	25.6 (21.5-29.9)	9.0%	0.35
90 to < 120	15.9[2] (8.2-26.7)	87.7 (83.9-90.9)	0.52 (0.47-0.56)	15.9 (12.6-19.7)	12.9%	0.81
120 to < 180	66.2 (53.7-77.2)	63.9 (58.8-68.9)	0.65 (0.59-0.71)	15.7 (12.4-19.4)	40.8%	2.60
180+	33.3[2] (9.9-65.1)	82.4 (78.5-85.9)	0.58[2] (0.44-0.72)	2.7 (1.4-4.8)	18.0%	6.68

1 – AUC, Area Under the operating Curve; IFA, Iron Folic Acid

2– Indicates uncertainty around this point estimate, as small number of true positive or negatives resulted in an estimate with a 95% confidence interval greater than fifteen percent.

Supplemental Figure 1

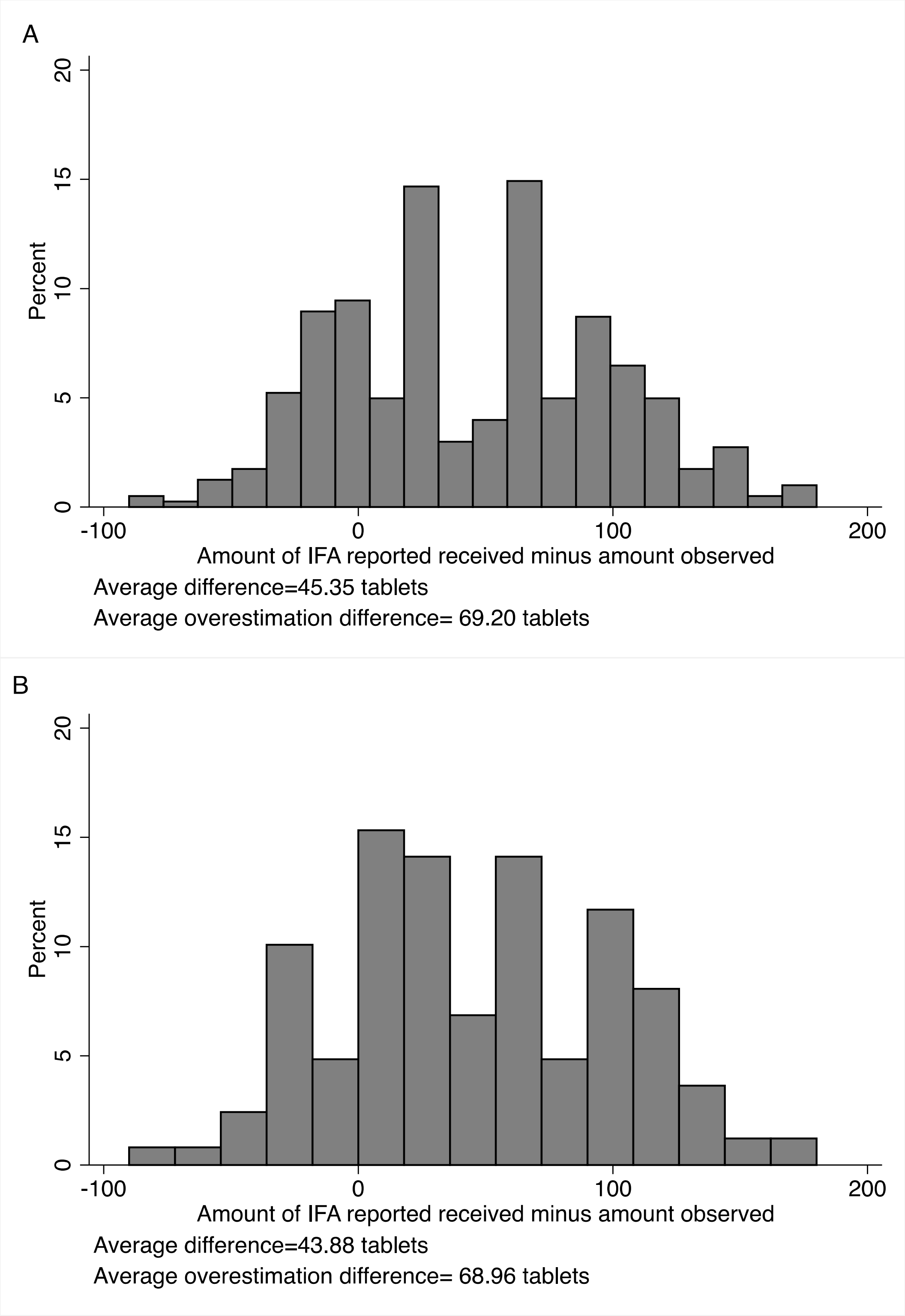

Distribution of the difference in IFA tablet counts reported received and observed received in (A) entire cohort (*N=402)* and B) sub-cohort (*N=248)*
